# Harnessing natural history collections for collaborative pandemic preparedness

**DOI:** 10.1093/biosci/biaf035

**Published:** 2025-06-02

**Authors:** Deborah L Paul, Cody W Thompson, Lizette Arroyo, Germán Botto Nuñez, Ivan Castro-Arellano, Jocelyn P Colella, Joseph A Cook, Michael V Cove, Jacqueline Dearborn, Noé U de la Sancha, Katrina Derieg, Jonathan L Dunnum, Adam W Ferguson, Kendall Fitzgerald, Courtney Foat, Lexi E Frank, Kurt Galbreath, Destiny F Gonzalez, Rebecca K Hawkins, Tommy Herrera, Alexander Hey, Andrew G Hope, Angela D Hornsby, Daniel Ibanez, Oliver Keller, Dianna Krejsa, Kailey Mahoney, Jason Malaney, Molly M McDonough, Thomas C McElrath, Verity Mathis, Teresa Mayfield-Meyer, Ilana Mosley, Nicté Ordóñez-Garza, Kendra Phelps, Roy N Platt, Josh Pletcher, Hilary H Rinsland, Charles E Rupprecht, Kelly A Speer, Laura Steger, Nathan Upham, Richard Allen White, Ben J Wiens, Litsa Wooten, Pamela S Soltis

**Affiliations:** University of Illinois, Champaign, Illinois, United States; University of Michigan, Ann Arbor, Michigan, United States; DePaul University, Chicago, Illinois, United States; Universidad de la República, Montevideo, Uruguay; Texas State University, San Marcos, Texas, United States; University of Kansas, Lawrence, Kansas, United States; University of New Mexico, Albuquerque, New Mexico, United States; North Carolina Museum of Natural Sciences, Raleigh, North Carolina, Unitd States; Smithsonian Institution, Washington, DC, United States; DePaul University, Chicago, Illinois, United States; University of Montana, Missoula, Montana, United States; University of New Mexico, Albuquerque, New Mexico, United States; Field Museum of Natural History, Chicago, Illinois, United States; Field Museum of Natural History, Chicago, Illinois, United States; University of Kansas, Lawrence, Kansas, United States; University of Minnesota, Saint Paul, Minnesota, United States; Northern Michigan University, Marquette, Michigan, United States; University of New Mexico, Albuquerque, New Mexico, United States; University of Oklahoma, Norman, Oklahoma, United States; University of California Berkeley, Berkeley, California, United States; University of Kansas, Lawrence, Kansas, United States; Kansas State University, Manhattan, Kansas, United States; University of Minnesota, Saint Paul, Minnesota, United States; University of Kansas, Lawrence, Kansas, United States; University of Michigan, Ann Arbor, Michigan, United States; University of Kansas, Lawrence, Kansas, United States; University of Utah, Salt Lake City, Utah, United States; New Mexico Museum of Natural History and Science, Albuquerque, New Mexico, United States; Chicago State University, Chicago, Illinois, United States; Illinois Natural History Survey, Champaign, Illinois, United States; University of Florida, Gainesville, Florida, United States; Albuquerque, New Mexico, United States; Texas A&M University, College Station, Texas, United States; University of Michigan, Ann Arbor, Michigan, United States; EcoHealth Alliance, New York, New York, United States; Texas Biomedical Research Institute, San Antonio, Texas, United States; Northern Michigan University, Marquette, Michigan, United States; Northern Michigan University, Marquette, Michigan, United States; Auburn University, Auburn, Alabama, United States; Northern Arizona University, Flagstaff, Arizona, United States; Arizona State University, Tempe, Arizona, United States; Arizona State University, Tempe, Arizona, United States; University of North Carolina, Charlotte, in Charlotte, North Carolina, United States; University of Kansas, Lawrence, Kansas, United States; Kansas State University, Manhattan, Kansas, United States; University of Florida, Gainesville, Florida, United States

Five years after the outbreak of the SARS-CoV-2 pandemic, we have the opportunity to gather lessons learned about needed infrastructure for predicting, understanding, and mitigating future zoonotic disease outbreaks. The world's natural history museums hold some 3 billion specimens that document life on Earth (Wheeler et al. 2012), each of which represents organismal diversity at a specific location and time and may carry a record of that organism's interactions with other species, from mutualists to pathogens, and its environment. In the context of disease biology, these specimens are an underused resource for understanding the geographic distributions of pathogens and their hosts, the origins and spread of disease, and the ecological conditions leading to spillover (e.g., Cook et al. 2020, Soltis et al. 2020, Colella et al. 2021, Thompson et al. 2021, Weems et al. 2021, Mabry et al. 2023). Moreover, the impacts of land-use alterations on the emergence and transmission of infectious diseases through complex relationships involving host stress and immunity, pathogen dynamics, and microbiome shifts (Botto Nuñez et al. 2019, Weems et al. 2021) and through increased exploitation and trade of wildlife can be tracked and interpreted using collections. We emphasize in the present article the potential role that collections could play if integrated into the One Health ecosystem of resources (see box 1). We focus in the present article on zoonotic diseases and relevant zoological collections; however, the same principles apply to collections and other threats to global biodiversity (e.g., Torres Lopez et al. 2024)—for example, herbarium specimens as resources for understanding plant pathogens and the spread of disease related to the world's food supply (e.g., Ristaino et al. 2021, Burbano and Gutaker 2023).

Despite their potential for contributing to pandemic preparedness, natural history collections are underused, and collaborations between the collections community and One Health efforts remain limited. To foster dialogue on the current and potential roles of museum collections in disease biology and how to move toward integrating this important infrastructure into mainstream pathobiology, two workshops funded by the National Science Foundation brought together participants from the museum collections community, One Health, pathobiology, bioinformatics, education, computer science, and art. In the present article, we highlight key issues and possible next steps that emerged from the workshops.

Box 1. **Select examples using natural history collections in disease biology.**

Bartonella: *Bartonella* is a bacterial genus known to cause human zoonoses such as Carrión's disease, cat-scratch fever, and trench fever (Jacomo et al. 2002). Using a targeted DNA enrichment approach, researchers identified *Bartonella* (and other zoonotic pathogens) in archived tissue samples from the mammal research collections housed in the Natural Sciences Research Laboratory at the Museum of Texas Tech University (TTU). *Bartonella* was found in 14 of 36 samples examined, consisting of rodents from Mexico and Senegal. This effort provides the framework for large-scale screening of pathogens in museum specimens, giving public health scientists a unique spatiotemporal perspective on zoonotic pathogen distribution, host associations, and evolution (Enabulele et al. 2023).

Hantaviruses: In 1993, a lethal hantavirus strain emerged in the southwestern United States, killing 10 individuals in the Four Corners region (Nichol et al. 1993). Described as Sin Nombre Virus (SNV), this was the second known hantavirus in the Western Hemisphere. Through traditional biological fieldwork (i.e., preservation of historical voucher specimens and associated frozen tissue samples) led by the Museum of Southwestern Biology at the University of New Mexico and TTU, researchers screened potential mammalian host species for the newly identified virus (i.e., SNV). Specimens showed that SNV had been circulating in the Southwest long before the 1993 outbreak, and the virus was detected in multiple host species (Yates et al. 2002; Goodfellow et al. 2025). In addition, researchers determined that hantavirus prevalence was cyclic and corresponded with El Niño climate cycles (Yates et al. 2002). Subsequent screening of museum tissue samples has significantly expanded the list of known hantaviruses and mammalian hosts, which now include moles, shrews, and bats (e.g., Arai et al. 2008, Yanagihara et al. 2014).

Mpox: Mpox (or monkeypox) is a viral zoonotic disease that is transmitted through animal-to-human and human-to-human contact (Reynolds et al. 2007). Originally identified from a colony of laboratory monkeys in Denmark (Magnus et al. 1959), Mpox is endemic to central and western Africa (Adetifa et al. 2023). Natural history collections have been critical to understanding the biology of the virus. For example, several orders of African mammals were collected and screened for Mpox to determine the extent of local infection. In total, six rodents and one elephant shrew were positive. These samples were identified and deposited in the Field Museum of Natural History (Doty et al. 2017). In addition, collections housed in the American Museum of Natural History (AMNH) and the Royal Museum for Central Africa were critical to identifying Mpox infections in a longitudinal series of African rope squirrel (*Funisciurus* sp.) specimens collected over a century (1899–1993). These data shifted the date of first detection of the virus by approximately a half century (Tiee et al. 2018).

Yellow fever: The Yellow Fever Service was an organization developed in the 1930s through a collaboration between the Brazilian Ministry of Education and the International Health Division of the Rockefeller Foundation (Soper 1977, Farley 2004). Through this collaboration, public health scientists from both organizations worked with biodiversity scientists from the AMNH to collect, voucher, and identify animal specimens to determine possible reservoirs of yellow fever (Gilmore 1943, Kumm and Laemmert 1950, de Oliveira and Franco 2005). Specimens were deposited in AMNH collections and used to determine the sylvatic transmission cycle of yellow fever virus (Soper et al. 1933, de Oliveira and Franco 2005), leading to the short-term eradication of the primary mosquito vector (*Aedes aegypti*) in Brazil (Laemmert et al. 1946, Löwy 1997). These specimens are still available to researchers via loan from the AMNH collections (Thompson et al. 2021).

## Interdisciplinary communication and network development

To ensure the data and expertise held in museums worldwide are effectively used to predict and prevent pandemics, these resources must be connected to disciplines and groups beyond the traditional museum user base. The data and expertise networks represented at our workshops do not yet organically intersect, and a reciprocal lack of shared knowledge and resources hampers progress (Kading and Kingston 2020).

Organizations and individuals not directly connected to collections often lack awareness of the data and samples available in museums, including ancillary data and expertise associated with each specimen (e.g., community membership) that can create new research opportunities. Building interdisciplinary networks will improve access to and use of both specimens and data in collections. Although data aggregators, such as GBIF (the Global Biodiversity Information Facility; gbif.org) and iDigBio (Integrated Digitized Biocollections; idigbio.org), host hundreds of millions of specimen records, work is needed to expand data mobilization to include information about species interactions, phenology, and other traits that can be gleaned from specimens (Balk et al. 2022). Improvements to data mobilization must also be reflected in the search interfaces of data aggregators, so that host–pathogen data are findable and accessible to biomedical researchers and disease ecologists.

Artists at our workshops illustrated presentations and conversations in real time (figure 1), capturing topics such as finding funding, extending vouchering, prioritizing longitudinal data, tracking microbiomes (e.g., wastewater monitoring), and improving database links (e.g., sample, voucher, microbiome data, host contextual information). Communication through art has the potential to link groups of scientists and to connect science with the public.

**Figure 1.**
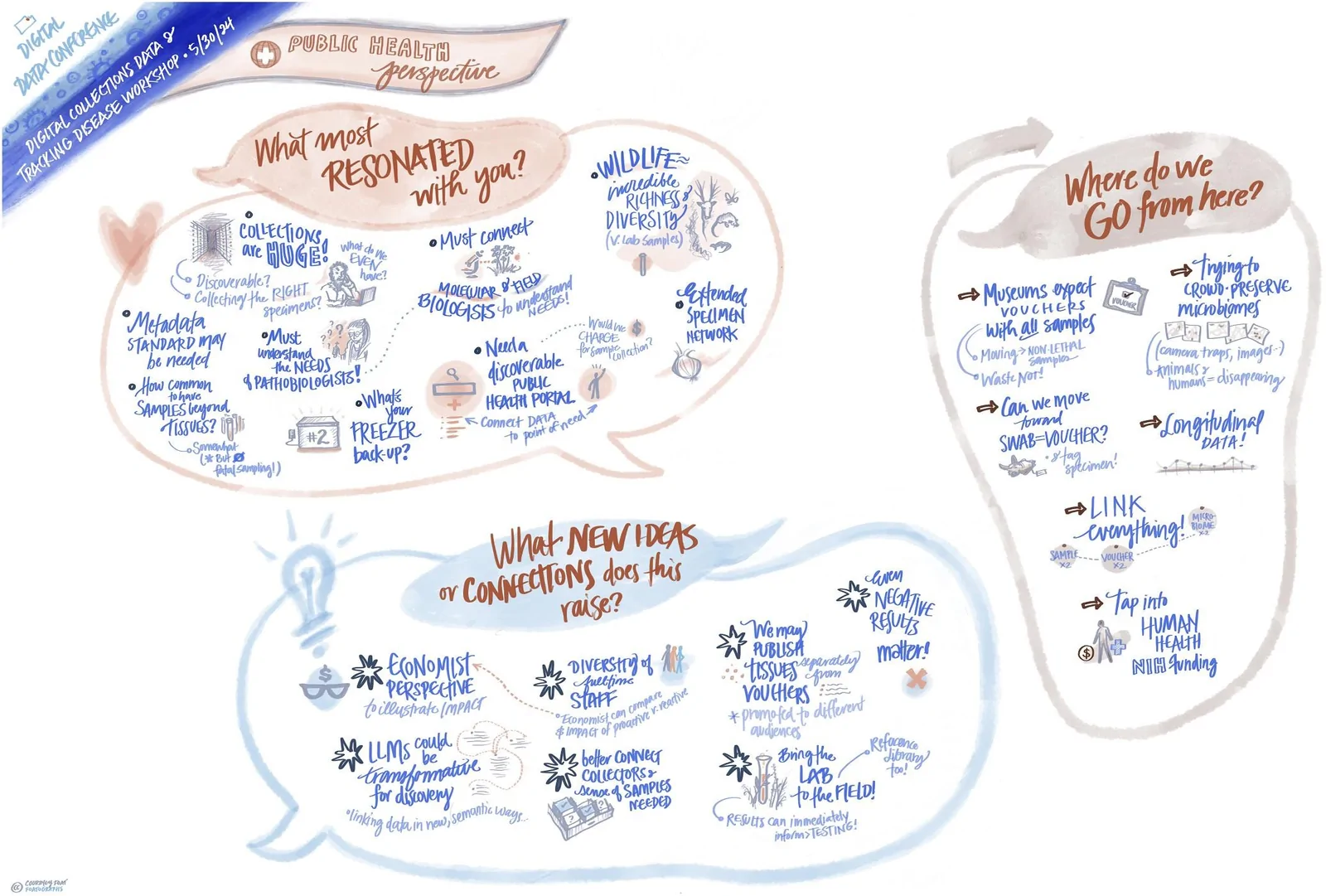
Artistic representation summarizing workshop discussions at the Digital Data Conference 8, held 30 May 2024, at the University of Kansas (Foat 2024).

## Expanding expertise through collaboration

Emerging from the workshops are three interconnected areas that need development for more effective strategies for pandemic preparedness: interdisciplinary awareness of collections data and expertise, the expansion of biosafety resources within the museum community, perhaps to institutions that currently lack biosafety facilities, and the development, sharing, and transfer of knowledge for effective collecting, storage, and use of samples. Notably, collections are not explicitly mentioned in the 2024 vector-borne disease national strategy (VBD) report (USDHS and USCDC 2024). Collections and associated staff are loosely included under the category of academic partners in the VBD public health strategy document. Museums can directly contribute to VBD goals but must improve the awareness within national and international agencies (e.g., the US Center for Disease Control and Prevention [CDC] or the National Institutes of Health [NIH]) regarding the relevance of collections in pandemic preparedness.

Networking between collections professionals and disease biologists could lead to new innovations. For example, White (2024), a virologist, argued that sample preservation techniques need to be developed that preserve molecular information and, specifically, RNA integrity longer term at room temperature, which has a lower energy and carbon footprint compared to ultracold freezers. White (2024) also emphasized the need to leverage proteomics and future protein-level sequencing techniques, because protein is more stable than RNA and could be useful for pathogen detection from archival samples, and noted that the design of new chemical preservatives could aid in the preservation and long-term stability of all biomolecules.

Additional monitoring of pathogens in wildlife is also needed (Colella et al. 2021). A pathogen with the lethality of smallpox (e.g., *Variola major*, 30%), infecting 50% of the US population (approximately 167 million), could result in approximately 50 million deaths (Machado et al. 2021), vastly affecting society. COVID-19 resulted in approximately 7 million deaths, millions more developed long-term symptoms, and it caused trillions of dollars in economic impacts (Knutson et al. 2023). These alarming statistics emphasize the need for collaboration between wildlife biologists and virologists (Johnson et al. 2015).

Botto Nuñez (2024) pointed out the need for taxonomists to protect economic, health, and natural resources as samples and specimens that are not accurately identified can cause deadly and expensive problems. Erroneous or incomplete determination of pathogen hosts coupled with incomplete field data and the absence of vouchers for checking identifications can lead to expenditures of time and money on misguided surveillance or control strategies (Guarino et al. 2013, Buckner et al. 2021, Novaes et al. 2021, Thompson et al. 2021, González and Botto 2024). Recognizing the important role that insects in particular play as disease-bearing vectors and the limited knowledge of the biology of insect hosts, the VBD group (USDHS and USCDC 2024) calls for strengthening the entomological workforce, noting the “lack of workforce diversity in the field of entomology” and concurrent opportunities to “implement programs to enrich the public health entomology workforce.” They emphasize that the lack of workforce diversity limits the ability of prevention and control agencies to address social, cultural, racial, ethnic, environmental, and community-based barriers. A globally diverse workforce is especially important as zoonotic diseases may arise anywhere on Earth, and scientists with local knowledge are best able to contribute. Moreover, given the diversity of disease vectors across many branches of the tree of life, the need for capacity development goes well beyond entomology and extends to all areas of taxonomic expertise to ensure accurate identification of vectors and appropriate implementation of surveillance and control programs.

## FAIR data (and specimen and sample) awareness and transparency

Awareness by pathobiologists and the public health sector of the data and expertise in natural history collections is only the beginning. We further note the need for (more) FAIR data (i.e., findable, accessible, interoperable, reusable). The VBD national strategy (USDHS and USCDC 2024) also identifies “lack of interconnected, quality data” as a major barrier, thereby highlighting an opportunity to “modernize data collection systems to increase learning and make data more readily available to decision-makers and the public.” Workshop participants similarly stressed the need to collect data more strategically and holistically (Galbreath et al. 2019, Thompson et al. 2021) by building new collections and aligning protocols and common data standards toward more rapid, networked data sharing (Hope et al. 2025). For example, connecting disparate sources of specimen-derived data requires special attention to how taxonomic names were used by different authors or collectors, which is often contained in difficult-to-access or undigitized literature archives (biological dark data; Upham et al. 2021, Phelps 2024). Therefore, efforts to digitize, extract, and link data from publications into digital accessible knowledge (Fawcett et al. 2022) are central to the work of sharing biodiversity knowledge bases with public health experts.

## An ethical view of sustainability

Expanding the reach and use of data, specimens, and expertise requires removing barriers to collecting specimens, supporting collections, and sharing data and materials. Adaptation is needed to meet regulations that protect curatorial staff, nature, and specimens, and doing so responsibly and with a long-term view will require an investment in museum infrastructure and workforce development. From sustaining collections and expertise, to sustaining planet-wide resources, strategic sampling at critical human–nature interfaces is needed, while simultaneously investing in resources to both preserve and make those samples and data available.

With the Nagoya Protocol on Access and Benefit-Sharing in mind (see www.cbd.int/abs/default.shtml), we note the need to share specimens, information, and other benefits quickly and effectively across the planet. At the same time, care is needed to determine how to do this in a constructive, equitable, and proactive way (Colella et al. 2023). Although the VBD is a domestic strategy, it acknowledges that it is “important to maintain complementary international capabilities that can help fight vector-borne global health security risks.” Clearly, an effective strategy cannot be limited to geopolitical or domain boundaries (Kading and Kingston 2020).

## Notable takeaways and next steps

Understanding the what, where, when, and why of host–pathogen interactions will be critical to anticipating whether and how humans may be affected (Johnson et al. 2015, Colella et al. 2021, Thompson et al. 2021, Cook 2024). Human lives and economies are at great risk, and the need for rapid and accurate taxonomic identification of vectors and hosts is immense (Olson and Juman 2024). Surveillance using a range of technologies in both the field and laboratory continues to improve (Frank et al. 2024), and efforts expanding and leveraging these advancements should be undertaken in concert with industry, agencies (e.g., the NIH and CDC), and nongovernmental organizations to help fund and coordinate capacity building and link infrastructure. Innovations in collection, storage, and preservation are needed to advance biomedical applications, and we urge the Society for the Preservation of Natural History Collections and other taxon-based organismal societies to work in concert with pathobiologists to optimize protocols. We encourage scientific societies, such as the Entomological Collections Network, to build collaborations that expand and extend the workforce to better study and track host–pathogen interactions.

We conclude by emphasizing the continued need to fund network development, workforce capacity development, collections infrastructure, fieldwork, and the digitization and mobilization of collections data relevant to pandemic preparation and prevention. The need to grow a network that considers biodiversity, evolution, ecology, and collections (Fenster et al. 2021) is obvious, and we see great potential in collaborations between this larger community of biodiversity scientists with pathobiologists and industry. The Museums and Emerging Pathogens in the Americas (MEPA) network, for example, is part of Project ECHO (Extensions for Community Healthcare Outcomes) and aims to better connect museum biorepositories with biomedicine. As of 2024, MEPA includes more than 300 participants (e.g., researchers, students, veterinarians, medical practitioners) representing more than 12 countries across the Americas (Colella et al. 2021). Similar and connected networks are needed across the world to grow local knowledge and to develop synergy to address global pandemics. We invite a conversation with the VBD national strategy leadership and members of the One Health network to see how our communities can align efforts, because there are clearly shared needs as we bring expertise and biodiversity infrastructure and informatics to this national, and international, conversation.

## Author Biography

Deborah L. Paul (dlpaul@illinois.edu) is affiliated with the University of Illinois, in Champaign, Illinois, in the United States. Cody W. Thompson, Oliver Keller, and Nicté Ordóñez-Garza are affiliated with the University of Michigan, in Ann Arbor, Michigan, in the United States. Lizette Arroyo and Noé U. de la Sancha are affiliated with DePaul University, in Chicago, Illinois, in the United States. Germán Botto Nuñez is affiliated with the Universidad de la República, in Montevideo, Uruguay. Ivan Castro-Arellano is affiliated with Texas State University, in San Marcos, Texas, in the United States. Jocelyn P. Colella, Courtney Foat, Alexander Hey, Daniel Ibanez IV, Dianna Krejsa, and Ben J. Wiens are affiliated with the University of Kansas, in Lawrence, Kansas, in the United States. Joseph A. Cook, Jonathan L. Dunnum, and Destiny F. Gonzalez are affiliated with the University of New Mexico, in Albuquerque, New Mexico, in the United States. Michael V. Cove is affiliated with the North Carolina Museum of Natural Sciences, in Raleigh, North Carolina, in the Unitd States. Jacqueline Dearborn is affiliated with the Smithsonian Institution, in Washington, DC, in the United States. Katrina Derieg is affiliated with the University of Montana, in Missoula, Montana, in the United States. Adam W. Ferguson and Kendall Fitzgerald are affiliated with the Field Museum of Natural History, in Chicago, Illinois, in the United States. Lexi E. Frank and Angela D. Hornsby are affiliated with the University of Minnesota, in Saint Paul, Minnesota, in the United States. Kurt Galbreath, Josh Pletcher, and Hilary H. Rinsland are affiliated with Northern Michigan University, in Marquette, Michigan, in the United States. Rebecca K. Hawkins is affiliated with the University of Oklahoma, in Norman, Oklahoma, in the United States. Tommy Herrera is affiliated with the University of California Berkeley, in Berkeley, California, in the United States. Andrew G. Hope and Litsa Wooten are affiliated with Kansas State University, in Manhattan, Kansas, in the United States. Kailey Mahoney is affiliated with the University of Utah, in Salt Lake City, Utah, in the United States. Jason Malaney is affiliated with the New Mexico Museum of Natural History and Science, in Albuquerque, New Mexico, in the United States. Molly M. McDonough is affiliated with Chicago State University, in Chicago, Illinois, in the United States. Thomas C. McElrath is affiliated with the Illinois Natural History Survey, in Champaign, Illinois, in the United States. Verity Mathis and Pamela S. Soltis are affiliated with the University of Florida, in Gainesville, Florida, in the United States. Teresa Mayfield-Meyer is affiliated with a biodiversity data consultant in Albuquerque, New Mexico, in the United States. Ilana Mosley is affiliated with the Texas A&M University, in College Station, Texas, in the United States. Kendra Phelps is affiliated with the EcoHealth Alliance, in New York, New York, in the United States. Roy N. Platt II is affiliated with the Texas Biomedical Research Institute, in San Antonio, Texas, in the United States. Charles E. Rupprecht is affiliated with Auburn University, in Auburn, Alabama, in the United States. Kelly A. Speer is affiliated with Northern Arizona University, in Flagstaff, Arizona, in the United States. Laura Steger and Nathan Upham are affiliated with Arizona State University, in Tempe, Arizona, in the United States. Richard Allen White III is affiliated with the University of North Carolina, Charlotte, in Charlotte, North Carolina, in the United States.
